# Fly-Ash Evaluation as Potential EOL Material Replacement of Cement in Pastes: Morpho-Structural and Physico-Chemical Properties Assessment

**DOI:** 10.3390/ma15093092

**Published:** 2022-04-24

**Authors:** Bogdan Stefan Vasile, Adrian-Ionut Nicoara, Vasile-Adrian Surdu, Vladimir Lucian Ene, Ionela Andreea Neacsu, Alexandra Elena Stoica, Ovidiu Oprea, Iulian Boerasu, Roxana Trusca, Mirijam Vrabec, Blaz Miklavic, Saso Sturm, Cleva Ow-Yang, Mehmet Ali Gulgun, Zeynep Basaran Bundur

**Affiliations:** 1 Department of Science and Engineering of Oxide Materials and Nanomaterials, Faculty of Applied Chemistry and Materials Science, University Politehnica of Bucharest, 011061 Bucharest, Romania; adrian.surdu@upb.ro (V.-A.S.); vladimir.ene@upb.ro (V.L.E.); ionela.neacsu@upb.ro (I.A.N.); alexandra.stoica@upb.ro (A.E.S.); iulianboerasu@gmail.com (I.B.); roxana.trusca@upb.ro (R.T.); 2 National Research Center for Micro and Nanomaterials, Faculty of Applied Chemistry and Materials Science, University Politehnica of Bucharest, 060042 Bucharest, Romania; ovidiu.oprea@upb.ro; 3 Department of Inorganic Chemistry, Physical Chemistry and Electrochemistry, Faculty of Applied Chemistry and Materials Science, University Politehnica of Bucharest, 011061 Bucharest, Romania; 4 Department of Geology, Faculty of Natural Sciences and Engineering, University of Ljubljana, Aškerčeva 12, 1000 Ljubljana, Slovenia; mirijam.vrabec@geo.ntf.uni-lj.si (M.V.); blaz.miklavic@geo.ntf.uni-lj.si (B.M.); 5 Department for Nanostructured Materials, Jozef Stefan Institute, Jamova cesta 39, 1000 Ljubljana, Slovenia; saso.sturm@ijs.si; 6 Materials Science and Nano-Engineering Program, Sabanci University, Orta Mahalle, Üniversite Caddesi No. 27, Tuzla–İstanbul 34956, Turkey; cleva.ow-yang@sabanciuniv.edu (C.O.-Y.); mehmet.gulgun@sabanciuniv.edu (M.A.G.); 7 Nanotechnology Application Center (SUNUM), Sabanci University, Orta Mahalle, Üniversite Caddesi No. 27, Tuzla–İstanbul 34956, Turkey; 8 Department of Civil Engineering, Ozyegin University, Nişantepe District, Orman Street, Çekmeköy, Istanbul 34794, Turkey; zeynep.basaran@ozyegin.edu.tr

**Keywords:** cement paste, EOL materials, SCM, recycling, circular economy, eco-friendly concrete

## Abstract

The main objective of the study was to produce alternative binder materials, obtained with low cost, low energy consumption, and low CO_2_ production, by regenerating end-of-life (EOL) materials from mineral deposits, to replace ordinary Portland cement (OPC). The materials analyzed were ash and slag from the Turceni thermal power plant deposit, Romania. These were initially examined for morphology, mineralogical composition, elemental composition, degree of crystallinity, and heating behavior, to determine their ability to be used as a potential source of supplementary cementitious materials (SCM) and to establish the activation and transformation temperature in the SCM. The in-situ pozzolanic behavior of commercial cement, as well as cement mixtures with different percentages of ash addition, were further observed. The mechanical resistance, water absorption, sorptivity capacity, resistance to alkali reactions (ASR), corrosion resistance, and resistance to reaction with sulfates were evaluated in this study using low-vacuum scanning electron microscopy.

## 1. Introduction

All over the world, the concrete industry plays an important role in the economy and development of a country, because concrete is an important material for infrastructure development. After water, concrete is the second most widely used material in the world and forms the backbone for a variety of buildings and civil engineering structures, such as highways, roads, bridges, water dams, buildings, skyscrapers, and even furniture [1]. Due to the increasing cement consumption, global production is expected to increase by more than 5 billion tons by 2030 [2].

Concrete consists of a mixture of cement, water, and several aggregates, including sand and gravel. Cement represents the key element in concrete production. The word cement originated in ancient Greece and Rome, where it was used to describe a masonry structure with a hard mass composed of lime and volcanic ash that reacted slowly in the presence of water. Due to its hydrating properties, constructional types of cement were also called hydraulic cement. Portland cement is the successor of hydraulic lime-based cement (lime-based mortars were widely used in construction until the second half of the 19th century when they were replaced by Portland cement) [3]. The well-known Portland cement receipt consists mainly of lime (calcium oxide, CaO) mixed with silica (silicon dioxide, SiO_2_) and alumina (aluminum oxide, Al_2_O_3_) [4,5]. The lime is obtained from a calcareous (lime-containing) raw material, and the other oxides are derived from an argillaceous (clayey) material. Various other raw materials such as silica sand, iron oxide (Fe_2_O_3_), and bauxite, Al(OH)_3_, are used to tailor the cement composition to a specific application.

Due to the massive global production and consumption of Portland cement, the cement industry is responsible for almost 10% of the world’s carbon dioxide emissions associated with anthropogenic activities [6] and consumes about 3% of the world’s energy [7]. It is reported that the production of 1 ton of cement releases about 1 ton of carbon dioxide gas into the atmosphere [8]. The pollution caused by cement production also includes the release of various other harmful gases such as nitrogen oxide and Sulphur dioxide [9,10]. Nevertheless, the cement industry is one of the major contributors to environmental pollution as a large amount of milling and grinding dust is released into the air. The resulting cement dust has adverse effects on plants, ecosystems, and human health [11]. In addition, the concrete industry is the largest consumer of fresh water and natural aggregates [7]. In recent years, the concrete industry has adopted different strategies to ensure the sustainability and protection of our planet. It is expected that these strategies combined with the development of concrete technology will enable the concrete industry to achieve net-zero emissions by 2050 [1].

Fortunately, in recent decades, the cement industry has identified some sustainable and eco-friendly alternatives to the conventional cement industry [12]. Compared to conventional OPC concrete, eco-friendly or green concrete can be classified as any concrete with less embodied energy and carbon [7]. In between, one of the most reliable alternatives is the use of supplementary cementitious materials (SCMs) [13,14,15,16,17,18]. SCMs have both pozzolanic and filler properties, which gives them the ability to increase the mechanical properties and durability of concrete. SCMs can be used as green substitutes as they are mainly waste products from various industries, and their use in concrete provides a way to effectively manage these waste products [7]. Some examples of SCMs are ground granulated blast furnace slag, silica fume, fly-ash, sugar cane bagasse ash, and rice husk ash [19].

Recent studies have demonstrated that fly-ash (FA) can be used as a mineral admixture in the cement industry to reduce the consumption of the most common calcareous raw materials [20]. Fly ash is the fine fraction of coal combustion by-products in power plants [19]. Fly-ash reacts with the calcium hydroxide produced during cement hydration reactions to form calcium silicate hydrate (CSH) gel, which contributes to the strength of concrete [21,22,23]. Substitution of fly-ash for structural concrete applications typically ranges from 15 to 35 wt.%, and substitutions up to 70 wt.% have been used for mass concrete in dams, walls, and roller compacted concrete pavements [24]. However, to achieve enhanced performance, an appropriate level of substitution must be used because durability issues, such as scaling carbonation and alkali-silica reactions, can be caused by the high amount of fly-ash in the concrete composition [7]. Another advantage of using fly-ash is its ability to delay early strength development [25,26,27,28]. ASTM C618 [29] specifies that the strength of mortars containing fly-ash must be at least 75% of the strength of ash-free mortar after either 7 or 28 days of curing. The particle size distribution of fly-ash can affect the compressive strength development, with ash having a higher proportion of smaller particles contributing more to the long-term strength [22]. The strength increase that fly-ash provides in well-cured concrete also enhances its structural resilience to repeated cycles of freezing and thawing if the concrete contains an appropriate amount (4–6 vol.%) of entrained air [30]. Usually, air-entraining agents (AEA) are added, consisting of aqueous mixtures of ionic or non-ionic surfactants [31]. Their non-polar end combines with small air bubbles and prevents them from coalescing and escaping from the mixture [31,32]. Fly-ash can increase the AEA requirement due to competitive adsorption on active carbon particles [31,33]. Accordingly, fly-ash may increase AEA demand if it contains a large carbon content. Maximizing the use of FA as SCM in the concrete industry seems to be an acceptable and feasible way to lead the industry in a more sustainable direction [19].

Many traditional investigation techniques fail when investigating hydrated cement in the wet state. Consequently, there is a lack of in situ knowledge of the geo-polymerization mechanism, as a result of a rapid chemical reaction [34]. The traditional Scanning Electron Microscopy (SEM) analytical technique fails in the study because it is a vacuum-based technique, which is not suitable for hydrated samples. Moreover, the traditional SEM requires sample preparation (dehydration, coating), which might affect and alter the native morphology of the sample surface. The ESEM (Environmental Scanning Electron Microscopy) technique allows the evaluation of hydrated or poorly conducting samples in situ without the need for special sample preparation. This technique includes a Peltier-cooled stage and a heating stage which allow good temperature control during the analysis. The temperature and relative humidity in the chamber can be controlled to simulate drying and wetting cycles on the sample. Zhang et al. reported the microstructure development of metakaolin-based geopolymers using in-situ and real-time ESEM studies [35]. Duchesne et al. also used the ESEM technique to monitor the reaction between the fly-ash and an alkaline activator in-situ [36]. The authors reported that the observed reaction was rapid, and the reaction products had a gel-like morphology. According to their ESEM observations, the fly-ash reaction is mainly limited to the surface of the particles and the microstructure can be described as a dense gel-like matrix with embedded fly ash particles.

In this work, the ash and slag from the Turceni thermal power plant deposit were initially investigated evaluated for morphology, mineralogical composition, elemental composition, degree of crystallinity, and heating behavior to determine their suitability as a potential source of SCM and also to establish the activation and transformation temperature in the SCM. The in-situ pozzolanic behavior of commercial cement (C0), as well as cement mixtures with 3 different percentages of ash addition were further observed. The cement pastes were also evaluated in terms of setting time, rheological properties, and workability. The microstructural changes of the cement paste with different SCM content were analyzed at different curing intervals (2, 7, 14, and 28 days, respectively). The mechanical resistance to compression, water absorption and sorption capacity, resistance to alkali reactions (ASR), corrosion resistance, and resistance to reaction with sulfates were evaluated in this study.

## 2. Experimental

### 2.1. Materials and Methods

The ash used in this article was collected from the deposit of the Turceni thermal power plant, which is located on the outskirts of Turceni, Gorj County, Romania. After characterization, the collected sample was dried at 60 °C for 2 days, ground, and sieved using a 45 m sieve and further used as an additional cementitious material.

The obtained cement pastes were based on a commercial cement CEM I (according to SR EN 197-1:2011), with or without ash addition, with the following compositions and notations: 0% (C0), 15% (C15), 30% (C30), 45% (C45). The pastes were obtained by adding the solid mixture and water in a 2:1 mass ratio and vigorous mechanical stirring until homogenization.

Afterward, the cement pastes were cast in a cylindrical mould with a diameter of 21 mm and a height of 16 mm. After stripping, the samples were stored in water at a temperature of 20 ± 2 °C and tested at intervals of 2, 7, 14, and 28 days, respectively according to SR EN 196-1:2016.

### 2.2. Characterization

Scanning electron microscopy (SEM) analysis of the ash powder and cement pastes after several days of curing was performed using an FEI Inspect F50 microscope coupled with an energy dispersive spectrometer (EDS) (ThermoFisher, Eindhoven, The Netherlands). The secondary electron was used at an accelerating voltage of 30 kV. The microstructure of the cement pastes and the in-situ reaction products formed during the first minutes to hours were studied using the Versa 3D Scanning Electron Microscope, which allows the adjustment of pressure, temperature, and humidity. Imaging was performed under low vacuum conditions (638 Pa) at a temperature of 2 °C and relative humidity of 90%, with a beam voltage of 30 kV, at a working distance of 9 mm (distance between the pole piece and the sample).

The phase composition and crystallinity of the initial ash, as well as in-situ evaluation for hydration of cement pastes, were studied by X-ray diffraction (XRD) using PANalytical Empyrean equipment. The equipment was used in transmission geometry and was equipped with a hybrid monochromator and a 1/2° divergent slit on the incident beam side and a programmable anti-scattering slit mounted on the PIXCel3D detector on the diffracted beam side. The sample was investigated in the range of 4–48° 2θ angle, with a step size of 0.026°, a time per step of 0.5 s, and a revolution speed of 0.5 s. Measurements were performed every 15 min for 24 h. During sample preparation, the water was mixed with the binder material and 10% aluminum oxide (corundum) in a ratio of 0.5:1. Aluminum oxide was added as a standard for crystallinity and was chosen because it does not interact with the binder mass. Reduction of X-ray diffraction data and full pattern fitting by the Rietveld method were performed using HighScorePlus 3.0.e software (PANalytical, Almelo, The Netherlands).

Thermal analysis was performed in an air atmosphere with a heating rate of 10 °C/min in a Netzsch TG 449C STA Jupiter instrument for the temperature range between room temperature and 1000 °C.

The determination of the setting time was carried out according to the standard SR EN 196-3:2017 using an automatic Vicatronic Vicat equipment from Matest (Matest, Treviolo, Italy). The determination of the rheological properties and therefore implicitly the workability of the binder pastes was performed by determining the dynamic viscosity. The dynamic viscosity was determined 5 min after mixing the solid components with water using a Brookfield Viscometer.

The mechanical resistance to compression was determined using a Shimadzu Autograph AGS-X 20 kN series (Shimadzu, Tokyo, Japan). To obtain more accurate results, all determinations were performed in triplicate.

Water absorption was performed on samples that had been matured under standardized conditions for 28 days. After this interval, the samples immersed in water were weighed and the mass was noted (*m_wet_*). After that, the specimens were dried at 110 °C for 24 h and then weighted again (*m_dry_*).

Absorption was calculated using the following formula:

$$Absorption=\frac{m_{wet}-m_{dry}}{m_{dry}}*100$$

*m_dry_* = dry sample mass (g);

*m_wet_* = sample mass after immersion (g).

Sorptivity was determined on cement cylinders with a diameter of 22 mm and a height of 17 mm. They were immersed in water to ensure water penetration only through the surface with which. All tested specimens were sealed by covering the sides with waterproof double-sided adhesive tape and then immersed in water to a height of 5 mm. Thus, the penetration of water is realized only by the capillary pressure. The amount of liquid absorbed by the specimen was determined for each time interval (from 10, 20, 30 min, up to 32 h) by weighing on an analytical balance.

The sorptivity was calculated using the following formula:

$$\mathrm{Sorptivity}=\frac{m_{wet}-m_{dry}}{A*\delta *t^{0.5}}$$

*m_dry_* = dry sample mass (g);

*m_wet_* = sample mass after immersion (g);

*A* = surface of the cylinder immersed in water (mm^2^);

δ = water density (g/cm^3^), *t* = time (min).

A 1 M sodium hydroxide solution (NaOH) was prepared to evaluate the resistance to alkali reaction (ASR), a 1 M solution of sulphuric acid (H_2_SO_4_) was prepared to evaluate the corrosion resistance and a solution of sodium sulfate (Na_2_SO_4_) with 5% concentration was prepared to evaluate the resistance to sulfate attack.

Thus, some of the specimens obtained above were removed from the water, dried, and weighed. They were then immersed in the previously prepared and weighed NaOH, H_2_SO_4_, or Na_2_SO_4_ solution and tested for their mechanical compressive strength tested at intervals of 7 and 14 days, respectively.

The mass change after immersion was calculated using the following formula:

$$Mass\;change=\frac{m_{immersion}-m_{initial}}{m_{initial}}*100$$

*m_initial_* = dry sample weight, before immersion (g);

*m_immersion_* = sample weight after immersion for 7 and 14 days, respectively (g).

Variations in mechanical compressive strength were calculated using the following formula:

$$Mecanical\;strength\;variation=\frac{R_{immersion}-R_{initial}}{R_{initial}}*100$$

*R_initial_* = mechanical compressive strength of the sample before immersion (MPa);

*R_immersion_* = mechanical compressive strength of the sample after immersion for 7 and 14 days, respectively (MPa).

## 3. Results and Discussion

### 3.1. Thermal Power Plant Ash Sample Characterization

**Figure 1:**
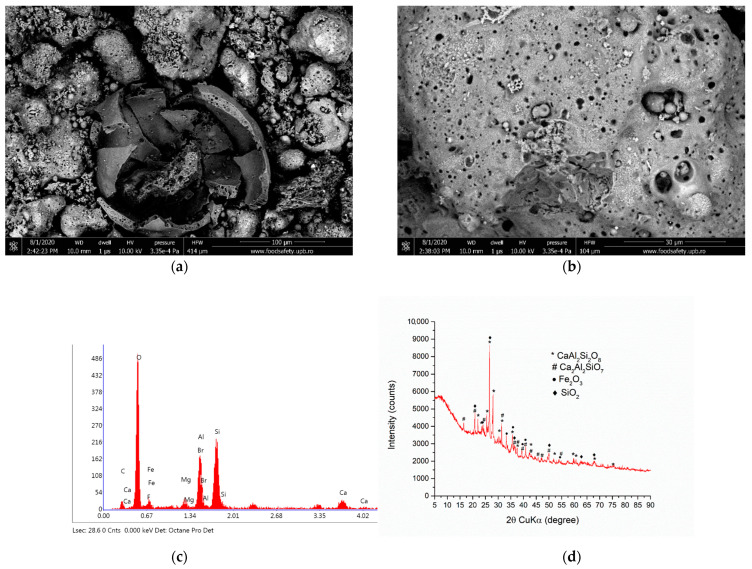
Secondary Electron Images (**a**,**b**), EDS spectra (**c**), and XRD pattern (**d**) of the ash sample.

The SEM images of the ash sample from the deposit of the Turceni Thermal Power Plant (Figure 1a,b) show a mixture of large aggregates with porous surfaces, globular shaped particles with diameters ranging from 10 to 300 µm, and some randomly distributed floc-like particles. Careful investigation of the globular particles revealed that they are mostly empty spheres with a rough surface and a porous wall up to 20 µm thick. In Figure 1c, the recorded EDS spectra show that Al, Fe, and Si are present as dominant elements in the studied samples, while the sample additionally contains Ca, K, and Mg. The crystalline phases consist of quartz (SiO_2_), hematite (Fe_2_O_3_), anorthite (CaAl_2_Si_2_O_8_), and gehlenite (Ca_2_Al_2_SiO_7_). The phase composition is presented below in Table 1, as determined by Rietveld’s refinement of the XRD pattern (Figure 1d). Phase analysis of the EOL sample shows a crystallinity degree of 20.32%.

**Figure 2:**
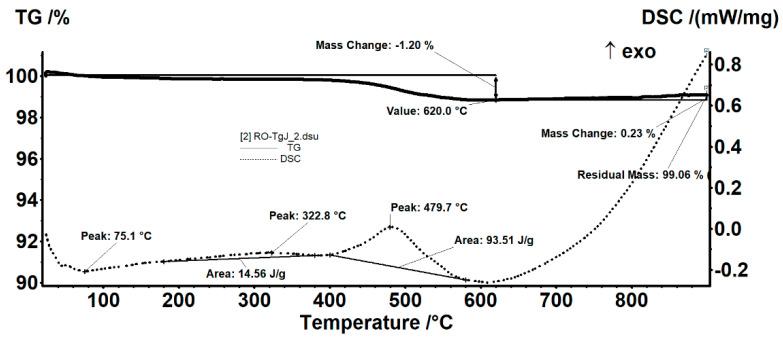
TG-DSC diagram between room temperature and 1000 °C in airflow. The heating rate was 10 °C/min.

In the low-temperature region (<100 °C), the samples lost absorbed moisture as can be seen from the endothermic effect with a minimum of 69.2 and 75.1 °C (see Figure 2). Between 180 °C and 380 °C, the sample slowly lost mass, with an accompanying wide and very weak exothermic effect with an area of 14–16 J/g. Oxidation of some carbon traces (probably in the amorphous content took place in the T-range of 400–600 °C. The maximum of this burning process was at 479.7 °C and its enthalpy was 79–93 J/g. The slight weight gain above 800 °C was probably due to oxidation of hematite changing the color of the powders to reddish-brown from the blackish-grey of the original ash.

### 3.2. The In-Situ Evaluation of Cement Pastes with SCM

Morphology changes during hydration of cement pastes were in situ ESEM monitored under variable humidity and temperature conditions. It is worth mentioning that the is situ ESEM dynamic experiments were performed on uncoated fresh wet cement mixture pastes. A gaseous secondary electron detector (GSED) was used. The sample holder was cooled by the Peltier plate. The heat in the device was dissipated by a continuous flow of water at 16 °C. Before imaging, mixtures of cement and ash were cooled to 2 °C for 15 min to stabilize their temperature. From the preparation of the pastes to their introduction into the microscope and the acquisition of images, there was a “dead” time during which the equipment was purged using an inert gas (N_2_). According to the pressure-humidity-temperature diagram of the liquid-vapor phase diagram for water, the expected pressure in the working chamber at a temperature of 2 °C and a humidity of 90% was estimated to be ~640 Pa. For about 10 min after mixing water and cement mixtures, possible reactions in the cement paste could not be monitored.

**Figure 3:**
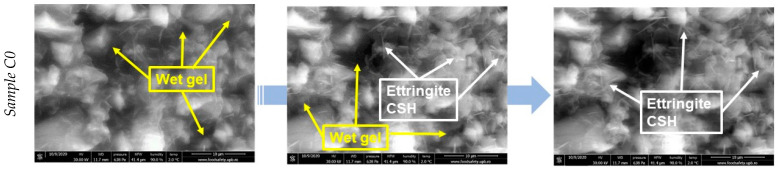

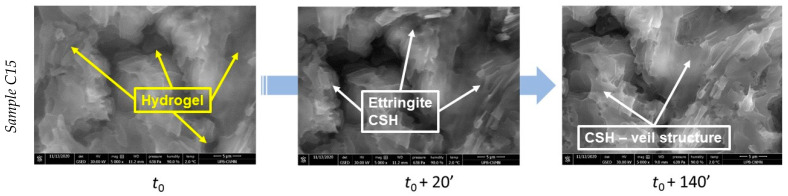
Scanning electron micrographs for C0 and C15 samples during hydration of the paste.

Each sample was monitored for a period of 3 to 6 h (depending on the specificity of the observed morphological changes) and the images were acquired every 10 min at magnifications of ×5000 and ×10,000. The morphologies for the sample with 100% cement (sample C0—reference) and the sample with 85% cement and 15% ash (sample C15) are shown in Figure 3. The legends show the time of acquisition, with the first image taken immediately after the “dead” time limit, called *t*_0_. In the micrographs of hydrated cement paste, we could observe the evolution of the first hydration products cement, with the formation of acicular structures of ettringite and CSH/CASH (calcium/aluminium silicate hydrates) [37,38]. Smaller particles are more reactive compared to larger ones. The hydration products tended to crystallize into cracks and pore spaces. A significant amount of cement hydration reactions appeared to have occurred during the “dead” period. The soft and fragile bride’s veil morphology of CSH appeared to have emerged from all surfaces.

**Figure 4:**
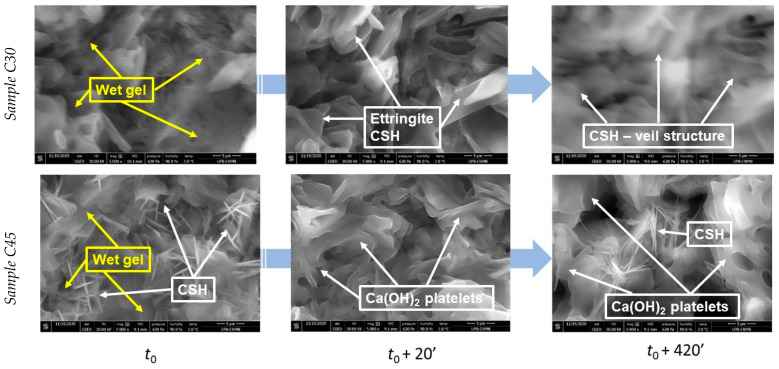
Scanning electron micrographs for sample C30 during hydration of the paste.

The addition of ash induced a delay in the hydration reactions, with the formation of prismatic sheet structures over time (Figure 4).

The hydration products appeared even more crystalline. The usual amorphous CSH veil structure appeared at later stages (420 min) of hydration. As the paste hydrates over time, the hydrogel resulting from the partial hydration of mineralogical compounds such as dicalcium silicate, tricalcium silicate, and tricalcium aluminate, covered the spaces between the particles of the specimen. At this highest loading content of ash into cement pastes (sample C45), the formed crystals appeared to have better-defined edges. Ca(OH)_2_ platelet structures could be captured after the “dead” time. The gel-like hydrosilicates covered the ash particles very well, and the needle structures responsible for the subsequent mechanical strengths of the binder material can be visualized in the advanced moments of hydration (*t*_0_ + 20′, *t*_0_ + 420′). The addition of ash in different proportions affects the hydration kinetics in the sense that some hydration reactions of the mineralogical binders are delayed with increasing ash content whereas Ca-initiated ones are accelerated. From a morphological point of view, gel-like hydrosilicates formed alongside Ca-based crystallite, which cover the ash-spheres and thus combine to form a compact binder mass.

**Figure 5:**
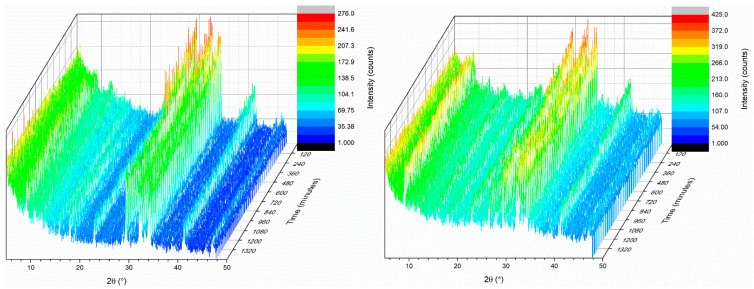
X-ray patterns of C0 (**left**) and C45 (**right**) samples, registered from *t*_0_ up to 24 h.

The XRD patterns obtained for the C0 cement paste and C45 sample, at the initial time point and after different time intervals from 15 min to 24 h, are presented in Figure 5. The intensity of the peaks decreases with time from the initial mixing of the constituents (*t*_0_), indicating a decrease in the degree of crystallinity (anhydrous phase content). The substitution of CEM I cement with fly-ash in a proportion of 45% did not drastically alter the phase evolution during hydration.

The phase analysis allowed the identification of the main compounds of C0 (C_3_S, C_2_S, C_3_A, C_2_F, and CaSO_4_∙0.67H_2_O), the compounds resulting from the reaction between C0 and water (ettringite, portlandite, and hydrocalumite) and the gypsum resulting from the hydration of basanite. The results of phase analysis are shown in Table 2 with a reference code, compound name, and chemical formula.

**Figure 6:**
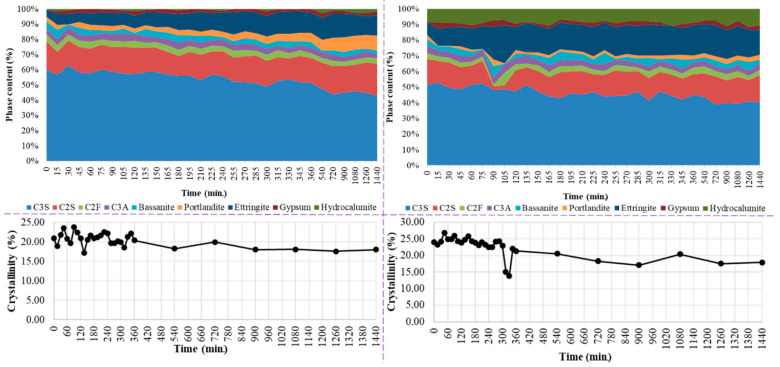
Phase analysis (**up**) and degree of crystallinity (**down**) of the C0 (**left**) and C45 (**right**) samples, from *t*_0_ up to 24 h.

The phase composition was determined from XRD data after fitting by the Rietveld refinement method. The evolution of the proportion of phases and the degree of crystallinity as a function of time are shown in Figure 6.

After the C0 cement paste is prepared, the first two crystalline phases were portlandite (3.3 wt.%) and ettringite (4.6 wt.%). A rapid decrease in the C_3_S (from 58.3 wt.% to 42.96 wt.%) and C_2_S (from 18.3 wt.% to 17.21 wt.%) content was observed from the composition in CEM I cement after 24 h, due to reaction with water. The percentage of portlandite increased from 3.3 wt.% to 4.4 wt.% after 4 h and 30 min, and after 24 h the percentage increased to 9.45 wt.%. A similar trend was observed in the case of ettringite. From inception of hydration to 4 h 30 min, the percentage increased to 13.34 wt.%. After 24 h ettringite percentage was 13.54 wt.%. In the beginning, after the preparation of the C45 binder paste, portlandite is formed in the proportion of 2.5 wt.% and ettringite 8.2 wt.%. Therefore, compared to the C0 binder paste, the reaction with water was faster in terms of initial ettringite formation and slower in terms of portlandite formation. Data analysis by different time intervals shows a trend of decreasing C_3_S and C_2_S content from 50.1 wt.% and 15.9 wt.% to 40.9 wt.% and 13.6 wt.%, respectively, after 24 h. This decrease is due to the reaction of the compounds with water to form portlandite and ettringite. The proportion of portlandite increased from 2.5% to 3.19% after 24 h. The slow increase in the proportion of portlandite may be related to its involvement in the pozzolanic reaction.

The evolution of the degree of crystallinity for C0 cement paste showed a decrease in the degree of crystallinity from 22.93 wt.% to 13.88 wt.% with the contact with water, which can be associated with the formation of gel-like hydrosilicates. After the initial reading, crystallinity revealed a decreasing trend up to 24 h. The distinct dip in the crystallinity ratio after 6 h of hydration was probably marking the end of the dormant period for the paste. The evolution of the degree of crystallinity for the C45 binder shows a decrease from an initial value of 19.94% to 18% after 24 h.

### 3.3. Flow Properties of SCM-Containing Pastes at Different Curing Intervals

**Figure 7:**
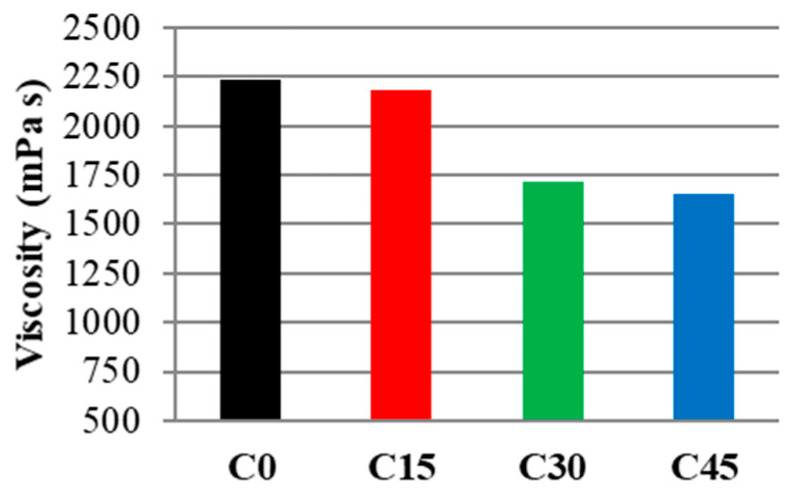
Sample viscosity after 5 min.

The initial setting time of the cement as measured with the Vicat needle test is the time elapsed between time zero and the time when the distance between the needle and the base plate is (6 ± 3 mm), measured with an accuracy of one minute. The results are presented in Table 3. Viscosity data in Figure 7 revealed that the addition of ash to the composition of the cement pastes made the paste less viscous, making the composition more workable. This behavior could be attributed to two possibilities: (i) at short curing times (5 min), the waste may accelerate the formation of gel structures, which implicitly could lead to an increase in the curing time. (ii) The waste ash could slow down the hydration reactions allowing excess water to act as a lubricant in the system.

**Figure 8:**
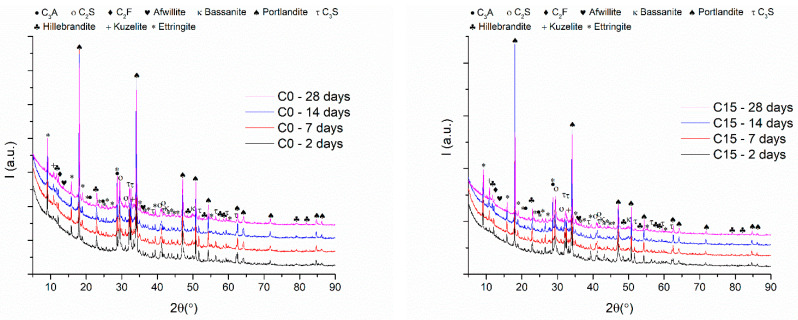

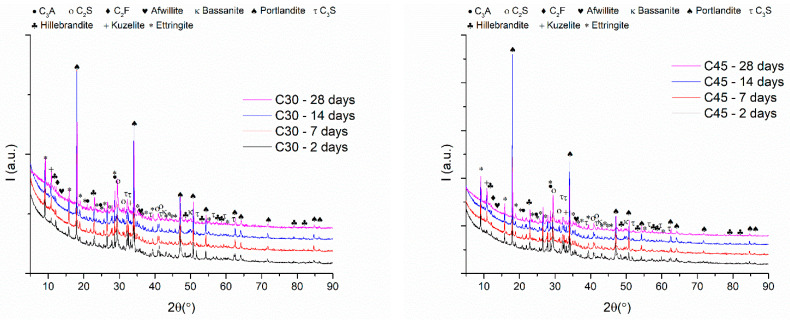
X-ray patterns of all samples after 2, 7, 14, and 28 days of curing time.

The results of XRD pattern analyses performed on all samples after 4 curing intervals are shown in Figure 8 and Table 4. They highlight the reaction of the mineralogical compounds of the CEM I cement (C_3_S, C_2_S, C_3_A, C_2_F) with water to form afwillite, hillebrandite, portlandite, kuzelite, and ettringite.

**Figure 9:**
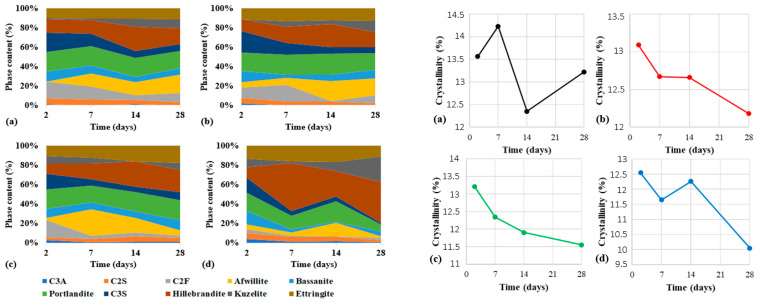
Phase analysis (**left**) and degree of crystallinity (**right**) of C0 (**a**), C15 (**b**), C30 (**c**), and C45 (**d**) samples after 2, 7, 14, and 28 days curing time.

The phase composition was determined from XRD data after fitting by the Rietveld refinement method. The evolution of the proportion of phases and the degree of crystallinity as a function of time are shown in Figure 9.

For the C0 sample, the analysis of the phases and plotting of the content as a function of time revealed the following aspects: (i) decrease in C_3_S content from 20.3% after 2 days to 6.9% after 28 days; (ii) increase in afwillite and hillebrandite content from 13.4% after 2 days to 34.9% after 28 days; (iii) decrease of the portlandite content from 20.2% after 2 days to 18% after 28 days; (iv) an almost stable content of ettringite content 10.2% after 2 days and 11.2% after 28 days. Moreover, the degree of crystallinity illustrated the cyclic formation and disappearance of amorphous and crystalline hydration products.

On the other hand, the same analysis performed on the C15 sample revealed: (i) a decrease in C_3_S content from 22.2% after 2 days to 5.8% after 28 days; (ii) an increase in afwillite and hillebrandite content from 17.7% after 2 days to 33.4% after 28 days; (iii) a decrease in portlandite content from 18.8% after 2 days to 17.3% after 28 days and (iv) an increase in ettringite content from 11% after 2 days to 12.7% after 28 days. In this case, the degree of crystallinity had a decreasing tendency over time up until 28 days of hydration in the blended cement pastes.

The analysis of the phases and plotting of the content as a function of time for the C30 sample highlighted the following aspects: (i) decrease in C_3_S content from 16.2% after 2 days to 8.1% after 28 days; (ii) increase in afwillite and hillebrandite content from 13.4% after 2 days to 43.1% after 7 days and a decrease to 28.6% after 28 days (iii) increase of ettringite content from 10.4% after 2 days to 17.8% after 28 days. Regarding the degree of crystallinity, a tendency to decrease over time is observed.

In the case of the C45 sample, the following modifications were registered: (i) a decrease in C_3_S content from 15.5% after 2 days to 2% after 28 days; (ii) an increase in afwillite and hillebrandite from 15.4% after 2 days to 44.10% after 28 days; (iii) a decrease in portlandite content from 18.8% after 2 days to 6.9% after 28 days and (iv) a decrease in ettringite content from 13.6% after 2 days to 11.1% after 28 days. A similar tendency to decrease crystallinity over time is observed in this case also. The content of calcium aluminates present in studied fly-ash which can react with the formation of C-A-S-H is low. As a result, the quantities of alumino-silicates formed in binder systems containing the fly-ash could not be identified by X-ray diffraction.

**Figure 10:**
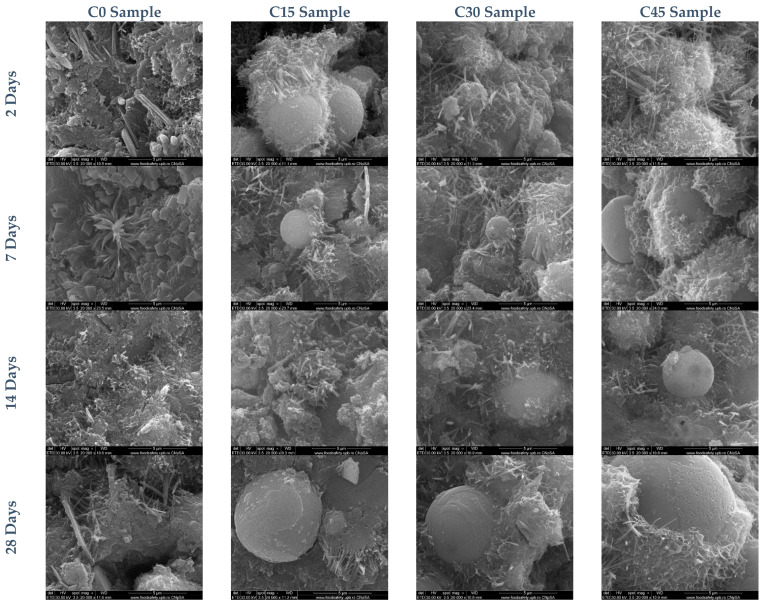
SEM images for Portland cement samples containing different ash proportions after 2, 7, 14, and 28 days of curing.

Scanning electron microscopy images for all samples after several days of curing are shown in Figure 10. After two days of hydration, the formation of characteristic hydration compounds with acicular shape (ettringite) and films of gel hydrosilicates on the surface of the clinker grains can be observed. With the addition of ash, a larger number of compounds are formed, especially on the surface of the spherical particles (cenospheres) that make up the ash. After 7 days, the samples show a similar microstructure, and the compounds resulting from hydration, which form mainly at the interface with the ash particles, are easily identifiable. A higher proportion of crystallized portlandite in the form of platelets was found in the samples without ash addition (C0).

After 14 days of hardening morphologies characteristic of gel hydrosilicates are obtained, the presence of which is confirmed by X-ray diffraction, the degree of crystallinity determined being 12%. The amount of portlandite formed increases compared to the previous test conditions but is lower compared to the standard sample (C0) at the same hydration conditions, which attests to the fact that the ash has a remarkable pozzolanic character. The standard test period (28 days) shows a microstructure characteristic of Portland cement. The gel hydrosilicates cover the ash particles very well and the needle structures can be visualized for each sample. XRD determinations indicate a decreasing degree of crystallinity from 14% for sample C0 to about 10% for sample C45.

**Figure 11:**
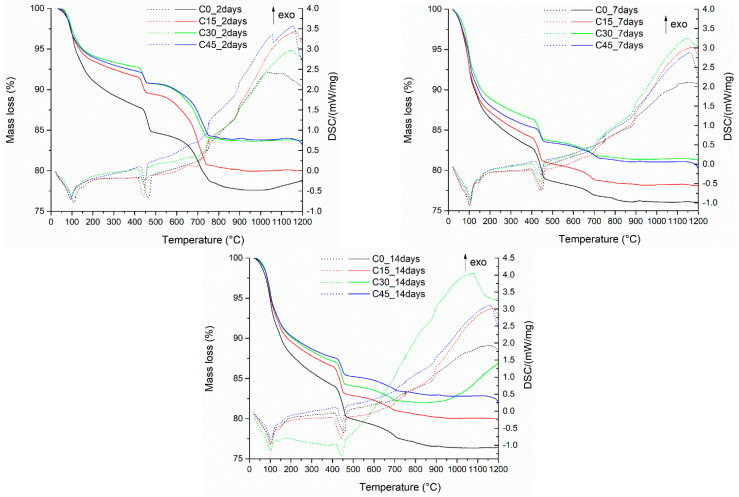
Differential thermal analysis of Portland cement samples containing waste in different proportions after 2, 7, and 14 days of hardening.

The thermal analysis performed on samples with different ash content (Figure 11) after two days of hardening shows the presence of two endothermic peaks with the maximum effect at temperatures around 102 °C, which can be attributed to the elimination of water present in the sample, and another one at a temperature of 450–460 °C, which can be attributed to the decomposition of portlandite. The mass loss of this last effect varies as a function of the amount of pozzolanic material added to the sample composition as follows: C0 has 2.83%, C15 has 1.91%, C30 has 1.80% and C45 has a weight loss of 1.28% measured at this temperature. This behavior indicates that Ca(OH)_2_ is consumed in the pozzolanic reaction caused by the presence of ash. The residual mass recorded at 1200 °C varies between 78–83% depending on the composition. After 7 days of continuous hydration, the mass losses associated with the presence of portlandite are recorded for each sample as follows: C0—3.69%, C15—2.61%, C30—2.28%, and C45—1.53%.

After 14 days of hydration, the mass losses associated with the presence of portlandite are further recorded for each sample as follows: C0—3.67%, C15—3.13%, C30—2.59%, and C45—1.98%, respectively. The increase in hydration time causes the formation of higher amounts of Ca(OH)_2_ resulting from the hydration process of the mineralogical compounds present in the Portland cement clinker. And in these cases, there is a decrease in the amounts of portlandite with increasing content of pozzolanic material in the composition of the analyzed samples.

**Figure 12:**
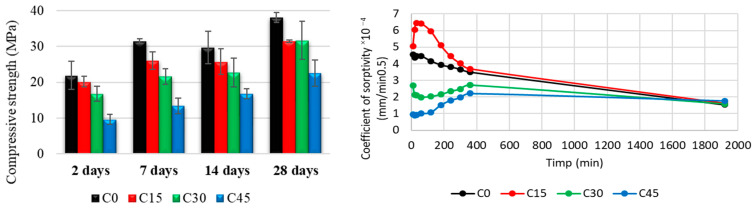
Mechanical compressive strength (**left**) and sorptivity (**right**) of waste containing Portland cement samples.

Comprehensive strength tests were carried out for the samples obtained. The results are shown in Figure 12, along with the sorptivity evaluation. The analysis of the mechanical behavior of the samples shows an increase in the mechanical strength after 28 days to about 38 MPa for the standard sample (C0), while the samples with 15% and 30% ash reach a maximum of 32 MPa in the same time interval. Increasing the ash content to 45% results in lower values of mechanical strength (22.5 MPa). It should be noted that after testing periods longer than 28 days, the optimal substitution ratio of Portland cement with ash is between 15 and 30%, with similar values for these compositions. As can be seen in Figure 12, as the ash content increases, the capillarity of the samples decreases. This phenomenon can be explained by better compactness of the samples, since ash with small dimensions (less than 45 µm) can act as a filler and fill the pores, resulting in the hardening.

**Figure 13:**
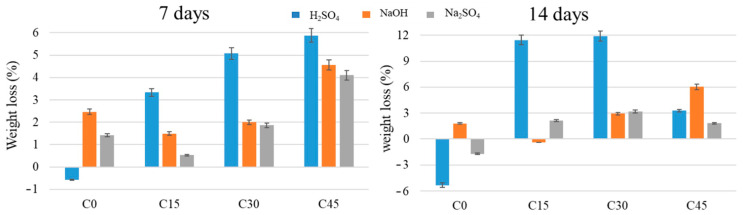
Mass changes of Portland cement samples containing waste in different proportions after immersion in corrosive media after 7 and 14 days.

After 7 days of immersion of the Portland cement specimens containing ash in NaOH, an increase in their mass is observed as the ash content is higher, except for the sample without waste addition (see Figure 13). There is also an increase in the mass of the specimens after 14 days of immersion, especially for the samples with a higher addition of waste (C30 and C45, respectively). After immersion in H_2_SO_4_, an increase in mass is observed as the amount of waste is higher, except for the sample with the addition of 45% waste (C45), where the mass increase is lower. After immersion in Na_2_SO_4_ of the waste-containing Portland cement specimens for 7 days and 14 days, respectively, an increase in their mass is observed as the content of waste is higher, except for the sample with the addition of 45% waste (C45), where the mass increase is lower after 14 days compared to 7 days. The sample without waste addition (C0) also shows a mass loss after 14 days.

**Figure 14:**
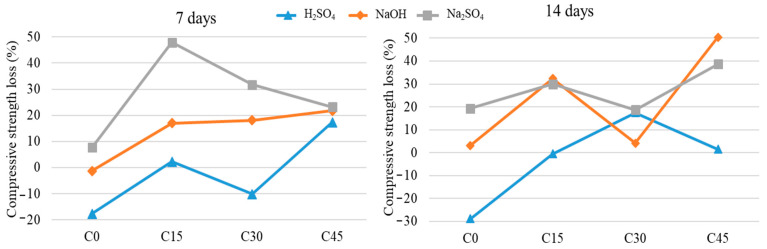
Changes in compressive strength of Portland cement samples containing waste in different proportions after immersion in corrosive media after 7 and 14 days, compared to the non-immersed samples.

Regarding the variation of the mechanical resistance to compression (Figure 14) for the samples immersed in NaOH, there are significant differences between the samples immersed for 7 and 14 days, respectively, and as a function of the percentage of waste added. Thus, the mechanical strength of the samples with an ash content of 45% increases by about 50% after 14 days of immersion, while lower ash percentages result in a smaller increase in mechanical strength. It should be noted that the sample with 30% ash content shows the lowest increase in mechanical strength after 14 days of immersion, which can be attributed to the homogeneity of the sample.

When immersed in H_2_SO_4_, significant differences are observed in the mechanical resistance to compression of the samples, depending on the immersion and the percentage of waste added. The sample with no waste added (C0) shows a loss of mechanical strength on compression of about 19% at 7 days and 29% after 14 days of immersion in 1 M sulphuric acid. The largest difference occurs in the sample with 30% added waste (C30), which shows an increase in values of about 18% after 14 days.

As for the variation of the mechanical resistance to compression for the samples immersed in Na_2_SO_4_, an increase in the values of mechanical resistance is observed for all the samples performed. Compared to the standard sample, which develops an increase in resistance of a maximum of 20% after immersion in sulfate solution, the ash samples show an improved behavior by developing better mechanical strengths, with variation values twice as high (about 40% compared to non-immersed samples with the same composition).

## 4. Conclusions

This paper proposes fly ash from a thermal power plant as an alternative binder material to replace ordinary Portland cement. After evaluating its suitability as a potential source of additional cementitious materials through numerous experimental methods, several conclusions arise. Hence, with the addition of ash to the cement paste (CEM I Portland cement), a delay in the hydration reaction was observed, with the formation of hardening structures at different times. As the hydration of the paste progressed, the gel hydrosilicates resulting from the partial hydration of the mineralogical compounds filled the intergranular spaces of the specimen. Replacing CEM I cement with ash did not change the hydration mechanism. The addition of ash in the composition of the samples was found to affect both the initial and the final setting time. Increasing the ash content to 45% resulted in lower mechanical strength values. Sustainability through the reuse of accessible economic and social resources is a way to achieve environmental balance while ensuring long-term development. The obtained results recommend the ash from the Turceni thermal power plant to be successfully used as SCM material to replace ordinary Portland cement.

## Figures and Tables

**Table 1:** Semi-quantitative analysis as determined by XRD.

No.	Ref. Code	Compound Name	Chemical Formula	SemiQuant [%]
1	01-075-8322	Quartz	SiO_2_	31
2	00-041-1486	Anorthite	CaAl_2_Si_2_O_8_	58
3	04-007-6009	Hematite	Fe_2_O_3_	4
4	04-015-7930	Gehlenite	Ca_2_Al_2_SiO_7_	7

**Table 2:** Composition of the C0 and C45 pastes determined from the XRD patterns.

No.	Reference Code	Compound Name	Chemical Formula
1	00-006-0495	C_3_A	Ca_3_Al_2_O_6_
2	00-018-0286	C_2_F	Ca_2_Fe_2_O_5_
3	00-020-0452	Hydrocalumite	Ca_2_(Al(OH)_6_)Cl(H_2_O)_2_
4	00-036-0617	Basanite	CaSO_4_·0.67H_2_O
5	00-055-0738	C_3_S	Ca_3_SiO_5_
6	04-006-9147	Portlandite	Ca(OH)_2_
7	04-011-5267	Ettringite	Ca_6_Al_2_(SO_4_)_3_(OH)_12_(H_2_O)_26_
8	04-013-6291	C_2_S	Ca_2_(SiO_4_)
9	00-006-0046	Gypsum	CaSO_4_·2H_2_O

**Table 3:** Setting time for prepared samples.

Sample Name	Initial Setting Time (hh:mm)	Final Setting Time (hh:mm)
C0	4:27	10:42
C15	4:56	12:23
C30	6:32	13:12
C45	6:20	12:36

**Table 4:** Identified crystalline phases for all samples.

No.	Reference Code	Compound Name	Chemical Formula
1	00-006-0495	C_3_A	Ca_3_Al_2_O_6_
2	00-009-0351	C_2_S	Ca_2_SiO_4_
3	00-018-0286	C_2_F	Ca_2_Fe_2_O_5_
4	00-029-0330	Afwillite	Ca_3_(SiO_3_OH)_2_·2H_2_O
5	00-036-0617	Bassanite	CaSO_4_·0.67H_2_O
6	00-044-1481	Portlandite	Ca(OH)_2_
7	00-055-0738	C_3_S	Ca_3_SiO_5_
8	04-012-1668	Hillebrandite	Ca_2_SiO_3_(OH)_2_
9	04-013-3303	Kuzelite	Ca_2_Al(SO_4_)0.5(OH)_6_(H_2_O)_3_
10	04-013-3691	Ettringite	Ca_6_Al_2_(SO_4_)_3_(OH)_12_(H_2_O)_26_

## Data Availability

The available data can be found at: https://www.micronanotech.ro/en/regenerarea-materiilor-prime-brute-si-a-produselor-aflate-la-sfarsit-de-viata-pentru-a-fi-reutilizate-in-cimenturi-betoane/ (19 March 2022).
